# Impact of COVID-19 on Nutrition, Food Security, and Dietary Diversity and Quality in Burkina Faso, Ethiopia and Nigeria

**DOI:** 10.4269/ajtmh.20-1617

**Published:** 2021-06-23

**Authors:** Isabel Madzorera, Abbas Ismail, Elena C. Hemler, Michelle L. Korte, Adedokun A. Olufemi, Dongqing Wang, Nega Assefa, Firehiwot Workneh, Bruno Lankoande, Angela Chukwu, Millogo Ourohire, Josiemer Mattei, Abdramane Soura, Yemane Berhane, Ali Sie, Ayoade Oduola, Wafaie W. Fawzi

**Affiliations:** 1 Department of Global Health and Population, Harvard T.H. Chan School of Public Health, Boston, Massachusetts;; 2 College of Natural and Mathematical Sciences, University of Dodoma, Dodoma, Tanzania;; 3 University of Ibadan Research Foundation, University of Ibadan, Ibadan, Nigeria;; 4 College of Health and Medical Sciences, School of Public Health, Haramaya University, Harar, Ethiopia;; 5 Department of Epidemiology and Biostatistics, Addis Continental Institute of Public Health, Addis Ababa, Ethiopia;; 6 Institut Supérieur des Sciences de la Population, Université de Ouagadougou, Ouagadougou, Burkina Faso;; 7 Department of Statistics, University of Ibadan, Ibadan, Nigeria;; 8 Nouna Health Research Center, Nouna, Burkina Faso;; 9 Department of Nutrition, Harvard T.H. Chan School of Public Health, Boston, Massachusetts;; 10 Department of Epidemiology, Harvard T.H. Chan School of Public Health, Harvard University, Boston, Massachusetts

## Abstract

Coronavirus disease 2019 (COVID-19) can have far-reaching consequences for developing countries through the combined effects of infection and mortality, and the mitigation measures that can impact food systems and diets. Using a mobile platform, this cross-sectional study evaluated the effect of COVID-19 on food prices and dietary quality for 1797 households in Nouna and Ouagadougou in Burkina Faso, Addis Ababa and Kersa in Ethiopia, and Lagos and Ibadan in Nigeria. We assessed the consumption of 20 food groups during the previous 7 days. The dietary diversity scores (DDS) and Prime Diet Quality Scores (PDQS) were used to assess dietary diversity and quality. We used generalized estimating equation (GEE) linear models to evaluate associations between price changes for staples, pulses, vegetables, fruits, and animal source foods (ASFs) with the DDS and PDQS PDQS. Most participants reported increasing prices of staples, pulses, fruits, vegetables and ASF, and ≥ 40% reported the decreased consumption of staples, legumes, and other vegetables and fruits. The DDS (except in Kersa and Ouagadougou) and PDQS were lower during the COVID-19 pandemic. Higher pulse prices were associated with lower DDS (estimate, −0.35; 95% confidence interval [CI], −0.74 to 0.03; *P* = 0.07) in the combined analysis and in Burkina Faso (estimate, −0.47; 95% CI, −0.82 to −0.11). Higher vegetable prices were positively associated with the DDS (estimate, 0.22; 95% CI, 0.08 to 0.37). Lower crop production (estimate, −0.54; 95% CI, −0.80 to −0.27) was associated with lower DDS. The price increases and worsening dietary diversity and quality call for social protection and other strategies to increase the availability and affordability of nutrient-rich foods during the COVID-19 pandemic and public health emergencies.

## INTRODUCTION

Coronavirus disease 2019 (COVID-19) caused by the novel severe acute respiratory syndrome coronavirus 2 (SARS-CoV-2) is a public health emergency^1^ that has significantly affected the world’s health and economy.^2^ Deaths from COVID-19 had surpassed 2.5 million and up to 116 million cases had been reported globally by March 7, 2021.^3^ The COVID-19 emergency could have far-reaching consequences for developing countries, where the combined effects of infection and mortality from COVID-19 itself, unintended consequences of corresponding mitigation measures, and the emerging global recession could impact nutrition and health.^4^ Sub-Saharan Africa (SSA) is vulnerable to the health, social, and economic impacts of COVID-19.^5^ This vulnerability is attributed to many factors, including poor health systems that hinder testing, timely detection, and access to services for the treatment of COVID-19.^6,7^ Furthermore, despite concerted efforts to improve access to food globally and in SSA, food insecurity remains a significant challenge, with the number of the undernourished increasing to approximately 690 million currently.^8,9^ Therefore, the impact of COVID-19 will exacerbate an already dire situation.

One of the main ways that COVID-19 has affected health and nutrition in SSA is through its disruption of the food supply chains. Countries in SSA have implemented far-reaching public health protection measures to address COVID-19, including partial and full lockdowns that are sometimes policed and last at least 1 month at a time or even longer in select countries. Additionally, countries have implemented social distancing, border closures, home confinements, and quarantine measures, and these could have impacted agriculture and food systems and the functioning of the health systems, resulting in social and economic disruptions.^9–11^ Many individuals in SSA and globally have lost employment because of the pandemic.^12^ Small-scale farming, which is the main source of livelihood in Africa, may have also been disrupted,^5^ with access to farming inputs and supplies limited by restrictions in mobility and factory closures. Furthermore, market closures have limited the availability of food. These factors have reduced the purchasing power of populations both directly and indirectly, undermined the capacity to produce and distribute food, and decreased physical access to food at the peak of the crisis.^10^ They also could have exacerbated food insecurity and poor nutrition for many in the region.^4,13^

Additionally, reports have suggested that access to nutrition services has been disrupted. The United Nations Children’s Fund (UNICEF) predicted a possible 30% reduction in the coverage of essential nutritional services in low-income and middle-income countries at the onset of the COVID-19 pandemic.^14^ Most schools have been closed because of lockdowns, resulting in disruptions to school feeding programs.^15^ Safety net programs, including community nutrition programs for children and pregnant and lactating women, have also been affected.^15^ This may have ultimately affected access to food.

Despite current efforts to control the COVID-19 pandemic, it is not clear when the effects of pandemic-related disruptions to people’s livelihoods, health, and food systems will end. It is unclear to what extent access to nutrition services, availability and access to food have been affected by COVID-19 in SSA; as well as their effects on food production, prices for staples and other food groups, and diet quality and nutrition in the region. Most African governments have tried to implement various measures to mitigate the impacts of the pandemic, including the distribution of foods to poor households. However, it has been projected that limited food production, a decline in family incomes, changes in the availability and prices of nutritious foods, and interruptions in nutrition and health services will lead to increased child malnutrition and mortality^13,16^ and potentially affect women and vulnerable households.

This study aimed to understand how COVID-19 and related disruptions have impacted food systems, food security, and access to and consumption of diverse and quality diets in Burkina Faso, Ethiopia and Nigeria. These sites are part of the Africa Research Implementation Science and Education (ARISE) Network, which comprises 21 member institutions from nine SSA countries that are centers of excellence in public health research. We selected these sites because they had existing data collection infrastructure, research capacity for producing high-quality survey data, and significant populations vulnerable to undernutrition. In the current study, we sought to understand the effects of COVID-19 on prices of key food groups and the predictors of dietary diversity and quality for men and women during the COVID-19 emergency in these locations.

## MATERIALS AND METHODS

### Setting.

This study was a cross-sectional study of six sites from three sub-Saharan African countries, Burkina Faso, Ethiopia, and Nigeria, that are part of the ARISE Network. The survey was conducted in Nouna (rural) and Ouagadougou (urban) in Burkina Faso, Kersa (rural) and Addis Ababa (urban) in Ethiopia, and Ibadan (rural) and Lagos (urban) in Nigeria. Ibadan has both urban and rural areas. For the purposes of the survey, we limited our study population to the rural local government areas in Ibadan. More detailed information on the geographical features and other demographic characteristics of selected ARISE network sites is provided elsewhere.^17,18^

### Study design.

Computer-assisted telephone interviews (CATIs) were used to collect survey data across the study sites, to assess knowledge and practices related to COVID-19 prevention and management, and to evaluate the far-reaching impacts of the outbreak on nutrition, health, and other domains. Data collection across all sites was conducted using telephone interviews between August and September 2020 in Burkina Faso, between October and November 2020 in Nigeria, between July and August 2020 in Kersa, and between August and September 2020 in Addis Ababa. Eligible households were randomly selected from sampling frames from the Health and Demographic Surveillance Systems (HDSS) in Burkina Faso and Ethiopia (Kersa), the National Living Standards Survey and telephone service providers in Nigeria, and a new household survey established in Ethiopia (Addis Ababa).

Briefly, the study aimed to recruit 1800 adults from Nigeria, Burkina Faso, and Ethiopia, with a maximum of 600 adults from each country (300 from each site). Resource and time constraints limited the selection of a larger sample. The study sites obtained telephone numbers for households selected from their sampling frames, with additional telephone numbers selected to allow for non-response or refusal to participate. We anticipated a response rate of 60% for the household questionnaire based on previous experience in the study areas. Therefore, the study randomly selected 500 households from each site. From each household, one adult 20 years or older was identified for the interview. Additional details regarding the study are provided elsewhere.^18^

A standardized questionnaire was developed and adopted as appropriate for each country and setting. Experienced translators at each site translated survey questionnaires into local languages. Study sites recruited male and female research assistants conversant with the local languages and with experience conducting health-related survey research in each local context. Research assistants received extensive training regarding conducting telephone surveys, obtaining verbal informed consent from respondents, and administering the survey questionnaires. Research assistants conducted the interviews from virtual call centers and were supervised by site supervisors and staff. They collected information regarding socio-demographic characteristics, including age, sex, head of household, household size, education, and occupation of respondents. Questions regarding knowledge, attitudes, practices, and perceptions of COVID-19 were also asked to respondents. Other information collected included mental health, healthcare utilization, water, sanitation, and hygiene (WASH), food pricing, food security, and dietary intake of respondents. On average, telephone interviews lasted 20 to 40 minutes.

### Exposure measures.

The exposure variables in the analysis were the changes in the prices of staples (e.g., maize, rice, cassava, and teff), pulses (e.g., beans, lentils, peas, and chickpeas), vegetables (e.g., spinach, cabbage, tomatoes, onions, and any locally available vegetables), fruits (e.g., bananas, oranges, and any locally available fruits), and animal source foods (ASFs; e.g., beef, chicken, dairy, eggs, and fish). Respondents were asked if the prices for each of those five food groups had been affected during the COVID-19 emergency. The price changes for these food groups were classified as no changes, increased, or decreased. We categorized the responses as binary for each food group, indicating an increase or decrease/no change in food prices.

### Outcomes.

The main study outcome was dietary diversity as measured using the Food and Agriculture Organization (FAO) Minimum Dietary Diversity for Women (MDD-W) index during the COVID-19 emergency. The secondary outcome was diet quality measured using the Prime Diet Quality Score (PDQS). Dietary intake was assessed for the study respondents (male and female) using a list of 20 commonly consumed food groups. The food lists were adopted to reflect local dietary sources for each of the food groups in each country. Respondents were asked to recall the number of days they consumed food from a list of 20 food groups during the past 7 days (during the COVID-19 pandemic) and during the period before the COVID-19 emergency.

### MDD-W.

Recalled foods were categorized into 10 food groups based on the MDD-W.^19^ The MDD-W has been evaluated as a measure of micronutrient adequacy among women.^19,20^ During this study, we applied the tool as a measure of dietary diversity for both men and women at the study sites. We grouped food groups consumed by respondents during the previous 7 days as follows: 1) grains, white roots and tubers, and plantains; 2) legumes (beans, peas and lentils); 3) nuts and seeds; 4) dairy; 5) meats, poultry and fish; 6) eggs; 7) vitamin A rich dark green vegetables; 8) other vitamin A rich fruits and vegetables; 9) other vegetables; and, 10) other fruits. During this analysis, which used 7-day recall, we divided weekly consumption of the food groups by seven to obtain a daily frequency of consumption. If a food group was eaten at least once each day during the previous week, then it was considered to contribute to the MDD-W (Supplemental Table 1). The dietary diversity score (DDS; range, 0–10) was computed as the number of food groups consumed, with a greater score indicating higher dietary diversity.

### Prime Diet Quality Score.

The Prime Diet Quality Score (PDQS) has been proposed as a measure of diet quality and has been associated with poor birth outcomes and pregnancy-related morbidities (gestational diabetes and hypertension) in previous studies.^21–23^ Foods consumed by respondents during the previous 7 days were classified into 20 food groups for the PDQS.^21,22^ Foods were classified into the following 14 healthy food groups: 1) dark green leafy vegetables, 2) other vitamin A rich vegetables and fruits, 3) cruciferous vegetables, 4) other vegetables, 5) whole citrus fruits, 6) other fruits, 7) fish, 8) eggs, 9) poultry, 10) legumes, 11) nuts, 12) dairy, 13) whole grains, and 14) liquid vegetable oils. In addition, we evaluated the consumption of 6 unhealthy food groups: 1) red meat, 2) processed meats, 3) refined grains and baked goods, 4) sugar-sweetened beverages (SSBs), 5) desserts and ice cream, and 6) potatoes, roots and tubers, based on criteria determined by previous studies.^21,22^ We made the following adaptations to the score for this study: we excluded the fried foods obtained away from home food group from the study; we assessed the consumption of dairy instead of low-fat dairy; a roots and tubers group was used in place of a potatoes group; and, we included red and orange fruits and vegetables in the other vitamin A-rich fruits and vegetables category instead of using a carrots food group. We also categorized maize flour-based products as refined grains.

Points were assigned for the consumption of healthy food groups as 0–1 serving/week (0 points), 2–3 servings/week (1 point) and ≥ 4 servings/week (2 points). Scoring for unhealthy food groups was assigned as 0–1 serving/week (2 points), 2–3 servings/week (1 point) and ≥ 4 servings/week (0 points) (Supplemental Table 1). Points for each food group were summed to obtain an overall score (range, 0–40), with a higher score indicating better diet quality.

### Statistical analysis.

Both descriptive and inferential statistics were used for the analysis. Descriptive statistics used frequencies for categorical variables and means and standard deviations (SDs) for continuous variables to summarize socio-demographic characteristics, nutrition and food security indicators by site. We also used frequencies to characterize household food security, food production and changes in food prices and diet quality. For inferential analyses, we used generalized estimating equation (GEE) linear models with exchangeable correlation,^24^ controlling for clustering by site, to evaluate the associations of increases in the prices of staples, pulses, vegetables, fruits and ASF with DDS and PDQS (secondary analysis) in cross-country analysis. We also conducted country-specific analyses for Burkina Faso, Ethiopia, and Nigeria. We used GEE linear models for these secondary analyses.

We considered the following as potential confounders: country and region; age (20–29 years, 30–39 years, 40 years or older); respondent sex (female/male); education (none or incomplete primary, primary school or incomplete secondary, secondary school or higher); household head (no/yes); household size; occupation (unemployed, farmer or casual labor, employed, student, self-employed or other); own crop production affected (unchanged, production has decreased, production has increased, not engaged in farming); and food insecurity (worried you would run out of food during the past month (no/yes); skipped a meal during the past month (no/yes); and, did not eat for a whole day during the past month (no/yes). We assessed food insecurity using three questions adapted from the Household Food Insecurity Access Scale, which is a tool that has been validated for measuring food security in developing countries.^25^

We selected potential confounders based on associations with the outcomes in univariate regression models at levels of *P* < 0.20. Statistical significance was established based on *P* < 0.05. The missing indicator method was used to adjust for missing confounder data in the analysis.^26^ Analyses were conducted using SAS 9.4 (Cary, NC).

### Ethics.

Ethical approval for the study was provided by the Institutional Review Board at the Harvard T.H. Chan School of Public Health and the ethical review boards in each country and at each site, including the Nouna Health Research Center Ethical Committee and National Ethics Committee in Burkina Faso, the Institutional Ethical Review Board of Addis Continental Institute of Public Health in Ethiopia, and the University of Ibadan Research Ethics Committee and National Health Research Ethics Committee in Nigeria. Verbal informed consent was obtained from all adult participants.

## RESULTS

We analyzed data from 1,797 households. Of these households, 297 were from Nouna and 300 were from Ouagadougou in Burkina Faso, 288 were from Addis Abba and 297 were from Kersa in Ethiopia, and 304 were from Ibadan and 311 were from Lagos in Nigeria. Of the study respondents, half (*N* = 898) resided in rural areas.

Table 1 describes the socio-demographic characteristics of the study population. Most respondents were male across sites, except in Addis Ababa and Ibadan, where 64.6% and 51.3% of respondents were female, respectively. The mean age (±SD) of respondents was 42.3 (±12.3) years, and respondents were, on average, older in Nouna, Ouagadougou and Ibadan, with more than 57% who were 40 years or older. In Burkina Faso and Kersa (Ethiopia), more than 70% of respondents had incomplete primary education or no formal education; however, in Nigeria, at least 68% of the respondents had secondary school education or higher. The mean ± SD household size was 6.4 ± 3.5 and as large as 9.9 ± 5.0 in Nouna. In rural areas of Kersa (86.5%) and Nouna (79.2%), most respondents were farmers or casual laborers in Ibadan (65.5%) and Lagos (52.2%), most respondents were self-employed or students. In Ouagadougou, most respondents were self-employed or students (46.7%), whereas in Addis Ababa they were unemployed (44.4%).

**Table 1:** Demographic characteristics of the study households in Burkina Faso, Ethiopia, and Nigeria (*N* = 1797)

		Burkina Faso		Ethiopia		Nigeria	
		Nouna	Ouagadougou	Kersa	Addis Ababa	Ibadan	Lagos
Location	Overall	Rural	Urban	Rural	Urban	Rural	Urban
N	1,797	297	300	297	288	304	311
Sociodemographic characteristics							
Female	658 (36.6)	35 (11.8)	96 (32.0)	66 (22.2)	186 (64.6)	156 (51.3)	119 (38.3)
Age of respondent (mean ± SD), years	42.3 ± 12.3	48.4 ± 13.1	47.3 ± 9.9	36.7 ± 7.6	38.8 ± 12.6	41.4 ± 12.2	40.8 ± 12.9
20–29	230 (13.5)	14 (4.7)	7 (2.3)	33 (11.1)	71 (24.7)	51 (18.7)	54 (21.1)
30–39	496 (29.0)	62 (20.9)	50 (16.7)	149 (50.2)	105 (36.4)	66 (24.3)	64 (25.0)
≥ 40	984 (57.5)	221 (74.4)	243 (81.0)	115 (38.7)	112 (38.9)	155 (57.0)	138 (53.9)
Education							
None or incomplete primary	778 (43.7)	229 (77.1)	213 (71.0)	221 (74.4)	102 (35.5)	11 (3.7)	2 (0.7)
Primary school or incomplete secondary	379 (21.3)	59 (19.9)	81 (27.0)	63 (21.2)	72 (25.1)	84 (28.2)	20 (6.6)
Secondary school or higher	623 (35.0)	9 (3.0)	6 (2.0)	13 (4.4)	113 (39.4)	203 (68.1)	279 (92.7)
Household							
Head of household	1,340 (74.6)	258 (86.9)	260 (86.7)	253 (85.2)	227 (78.8)	154 (50.7)	188 (60.5)
Household size	6.4 (± 3.5)	9.9 ± 5.0	7.3 ± 3.0	7.0 ± 2.2	4.2 ± 1.7	5.3 ± 2.5	4.9 ± 2.2
Occupation							
Unemployed	203 (11.9)	7 (2.4)	58 (19.3)	26 (8.8)	100 (44.4)	4 (1.3)	8 (2.8)
Farmer or casual labor	565 (33.2)	229 (79.2)	52 (17.3)	257 (86.5)	0 (0.0)	19 (6.3)	8 (2.8)
Employed	300 (17.6)	13 (4.5)	50 (16.7)	3 (1.0)	30 (13.3)	81 (26.9)	123 (42.3)
Student, self-employed, or other	635 (37.3)	40 (13.8)	140 (46.7)	11 (3.7)	95 (42.2)	197 (65.5)	152 (52.2)
Access to safe and clean water for preparing food	1,568 (87.6)	256 (88.0)	239 (79.7)	197 (66.3)	277 (96.2)	290 (95.7)	309 (99.4)
Access to soap for handwashing	1,748 (98.4)	284 (97.6)	293 (98)	291 (99.0)	286 (99.7)	288 (96.6)	306 (99.4)
Access to water for handwashing	1,771 (98.8)	290 (98.6)	295 (98.3)	296 (99.7)	283 (98.3)	299 (99.0)	308 (99.0)
Provision of school meals for children stopped during COVID-19	584 (88.9)	208 (94.1)	132 (89.8)	69 (100)	132 (88)	31 (67.4)	12 (50)

Data shown as mean ± standard deviation (SD) or N (%).

### Food prices and food security.

Table 2 describes the food security characteristics of households in the study population. At all sites, most households reported that the prices of staples, pulses, fruits, vegetables, and ASFs had increased since the start of COVID-19, with the overall highest reported increases for staple grains (90.1%). Up to 98.7% of the households in Lagos and 71.0% (lowest) of households in Nouna reported an increase in staple grain prices. Up to 98.0% of households in Kersa and 69.6% (lowest) in Nouna reported price increases in pulses. For vegetables, the highest prevalence of price increases was reported in Lagos (98.0%) and the lowest prevalence was reported in Nouna (61.2%). However, for fruits, the highest prevalence of price increases was 97.0% in Lagos and the lowest prevalence was 59.7% in Addis Ababa. Finally, for ASFs, the highest prevalence of price increases was reported in Lagos (98.3%) and the lowest was reported in Nouna (61.2%).

**Table 2:** Description of food security characteristics for study households in Burkina Faso, Ethiopia, and Nigeria (*N* = 1797)

		Burkina Faso		Ethiopia		Nigeria	
	Overall	Nouna	Ouagadougou	Kersa	Addis	Ibadan	Lagos
N		297	300	297	288	304	311
Staple prices							
Unchanged	144 (8.5)	72 (26.5)	34 (12.1)	6 (2.0)	9 (3.6)	20 (6.7)	3 (1.0)
Decreased	24 (1.4)	7 (2.6)	0 (0.0)	1 (0.4)	8 (3.17)	7 (2.4)	1 (0.3)
Increased	1,533 (90.1)	193 (71.0)	248 (87.9)	288 (97.6)	235 (93.3)	270 (90.9)	299 (98.7)
Pulse prices							
Unchanged	175 (10.5)	75 (27.5)	52 (19.3)	6 (2.0)	19 (7.9)	18 (6.0)	5 (1.6)
Decreased	22 (1.3)	8 (2.9)	3 (1.1)	0 (0.0)	3 (1.3)	6 (2.0)	2 (0.7)
Increased	1,473 (88.2)	190 (69.6)	214 (79.6)	286 (98.0)	218 (90.8)	274 (92.0)	291 (97.7)
Fruits prices							
Unchanged	257 (15.6)	94 (36.7)	62 (23.9)	11 (3.8)	61 (24.6)	21 (7.1)	8 (2.7)
Decreased	56 (3.4)	9 (3.5)	1 (0.4)	1 (0.4)	39 (15.7)	5 (1.7)	1 (0.3)
Increased	1,335 (81.0)	153 (59.8)	197 (75.8)	276 (95.8)	148 (59.7)	270 (91.2)	291 (97.0)
Vegetables prices							
Unchanged	188 (11.1)	89 (34.5)	44 (15.7)	10 (3.4)	18 (6.7)	22 (7.4)	5 (1.6)
Decreased	64 (3.8)	11 (4.3)	5 (1.8)	2 (0.7)	39 (14.6)	6 (2.0)	1 (0.3)
Increased	1,449 (85.1)	158 (61.2)	232 (82.5)	279 (95.9)	211 (75.7)	271 (90.6)	298 (98.0)
Animal source foods prices							
Unchanged	207 (12.2)	77 (28.7)	55 (20.2)	11 (3.7)	43 (16.2)	18 (6.0)	3 (1.0)
Decreased	51 (3.0)	27 (10.1)	0 (0.0)	0 (0.0)	15 (5.6)	7 (2.4)	2 (0.7)
Increased	1,440 (84.8)	164 (61.2)	217 (79.8)	283 (96.3)	208 (78.2)	272 (91.6)	296 (98.3)
Food security							
Worried you would run out of food (past month)	1,129 (67.8)	127 (44.4)	193 (64.3)	236 (79.5)	154 (54.2)	220 (73.8)	199 (65.3)
Skipped a meal (past month)	604 (34.0)	39 (13.7)	72 (24.0)	41 (13.9)	57 (19.9)	213 (70.3)	182 (59.1)
Went without eating for a whole day (past month)	267 (15.1)	27 (9.6)	35 (11.7)	9 (3.0)	44 (15.3)	79 (26.3)	73 (23.7)
Social protection							
Assistance in cash or other means (local government, not-for-profit organization)	220 (12.3)	12 (4.1)	70 (23.3)	43 (14.5)	44 (15.3)	28 (9.2)	23 (7.4)
Cash	47 (2.6)	2 (0.7)	15 (5.0)	12 (4.0)	6 (2.1)	5 (1.6)	7 (2.3)
Food	134 (7.5)	7 (2.4)	15 (5.0)	31 (10.4)	37 (12.9)	27 (8.9)	17 (5.5)
School meals	5 (0.3)	2 (0.7)	0 (0.0)	0 (0.0)	1 (0.4)	1 (0.3)	1 (0.3)
Other	55 (3.1)	1 (0.3)	47 (15.7)	1 (0.3)	4 (1.4)	1 (0.3)	1 (0.3)
Own crop production was affected by the COVID-19 emergency							
Unchanged	394 (51.8)	124 (43.7)	80 (87.9)	142 (49.3)	3 (60.0)	42 (56.8)	3 (15.8)
Production has decreased	246 (32.3)	77 (27.1)	10 (11.0)	129 (44.8)	1 (20.0)	23 (31.1)	6 (31.6)
Production has increased	121 (15.9)	83 (29.2)	1 (1.1)	17 (5.9)	1 (20.0)	9 (12.2)	10 (52.6)

COVID-19 = coronavirus disease 2019. Data shown are N (%). Crop production frequencies shown are only among those participating in farming.

Overall, 67.8% of the households reported worrying about running out of food, with the highest prevalence reported in Kersa (79.5%) and the lowest prevalence reported in Nouna (44.4%). Approximately 34.0% of households overall and as many as 70.3% in Ibadan reported skipping a meal during the previous month. Finally, 15.1% of the households reported not eating for an entire day, with the highest prevalence in Ibadan (26.3%) and the lowest prevalence in Kersa (3.0%). Only 12.3% of households reported receiving social assistance to cope with COVID-19, with the highest prevalence in Ouagadougou (23.3%) and the lowest prevalence in Nouna (4.1%). Food assistance was the most common type of assistance provided; it was received by 7.5% of the study population. Farming was most commonly practiced in rural communities in Nouna and Kersa. Up to 44.8% of the households in Kersa indicated that crop production had decreased during the COVID-19 pandemic.

### Dietary diversity and diet quality.

The mean DDS (±SD) were low in Nouna (1.3 ± 1.4), Ouagadougou (1.8 ± 1.4), Kersa (2.3 ± 1.0), Addis Ababa, (1.9 ± 1.0), Ibadan (2.0 ± 1.8), and Lagos (2.2 ± 1.8). The median (IQR) PDQS were also low in Nouna (19; 17–21), Ouagadougou (19; 16–21), Kersa (18; 16–20), Addis Ababa (17; 15–19), Ibadan (18; 15–21), and Lagos (20; 16–23); the maximum PDQS was 40 during the COVID-19 pandemic.

### DDS.

**Figure 1.**
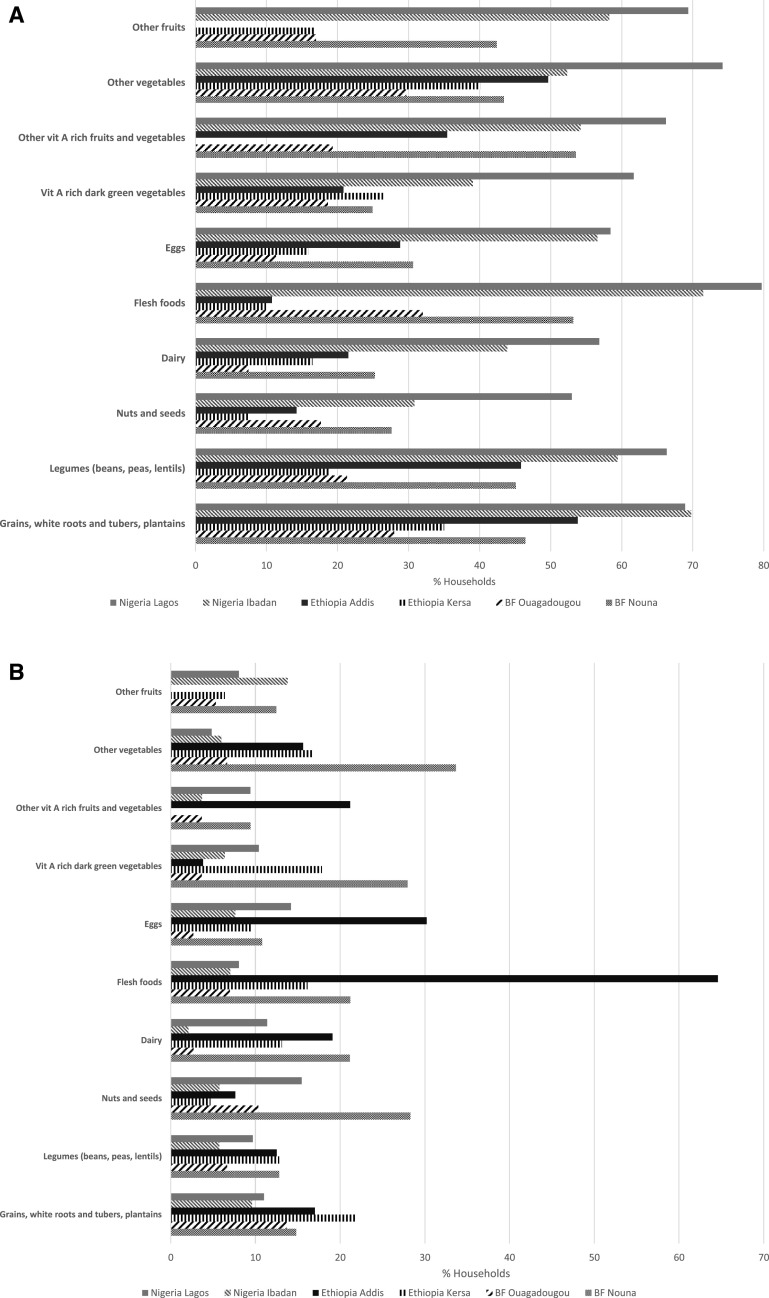
Changes in consumption of Minimum Dietary Diversity for Women (MDD-W) food groups before and during the coronavirus disease 2019 (COVID-19) pandemic in Burkina Faso, Ethiopia, and Nigeria. (**A**) Decreasing consumption of dietary diversity score (DDS) food groups in Burkina Faso, Ethiopia, and Nigeria during the COVID-19 pandemic. (**B**) Increasing consumption of DDS food groups in Burkina Faso, Ethiopia, and Nigeria during the COVID-19 pandemic.

Figure 1 shows the consumption of DDS food groups across the study sites. Regarding the consumption of DDS food groups overall, at least 50% of the households reported decreased consumption of staples and at least 40% reported decreased consumption of legumes, meats, poultry and fish, other vitamin A-rich vegetables, other vegetables, and other fruits (results not shown).

In Lagos, at least half of the study households reported reductions in the consumption of all DDS food groups (Figure 1A). In Ibadan, similar reductions were reported for all groups except nuts and seeds, dairy, and vitamin A-rich dark green vegetables (30–44%). In Kersa, at least 35% of households reported decreased consumption of staples and other vegetables. In Addis Ababa, respondents reported decreased consumption of staples (53.8%), other vegetables (49.7%), and legumes (45.8%). However, respondents at the same site reported increased consumption of meats, poultry and fish (64.6%), and eggs (30.2%) (Figure 1B). Finally, in Nouna, more than 50% of respondents reported decreased consumption of meats, poultry and fish, and other vitamin A-rich fruits and vegetables; however, in Ouagadougou, the consumption of food groups was unchanged for most households during the COVID-19 pandemic.

### PDQS.

The consumption of healthy and unhealthy food groups for the PDQS is presented in Figure 2. Across all sites, more than one-third of households reported that consumption of healthy (Vitamin-A rich vegetables and fruits, legumes, cruciferous vegetables, citrus and other fruits, poultry, legumes, and eggs) and unhealthy food groups (red meat, potato, roots and tubers, sugar-sweetened beverages, and refined grains) had decreased compared with that during pre-COVID-19 times (results not shown).

**Figure 2A.**
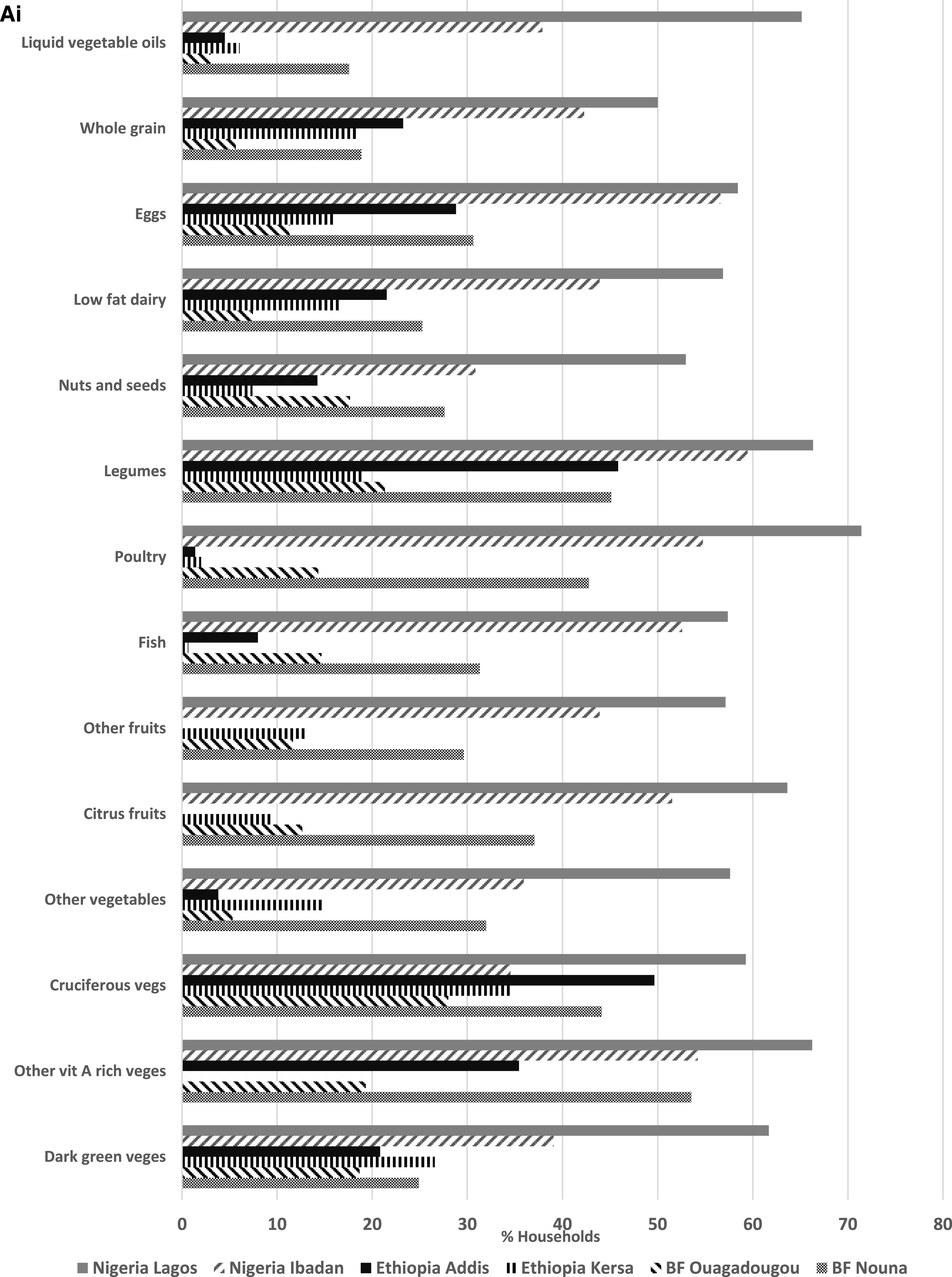

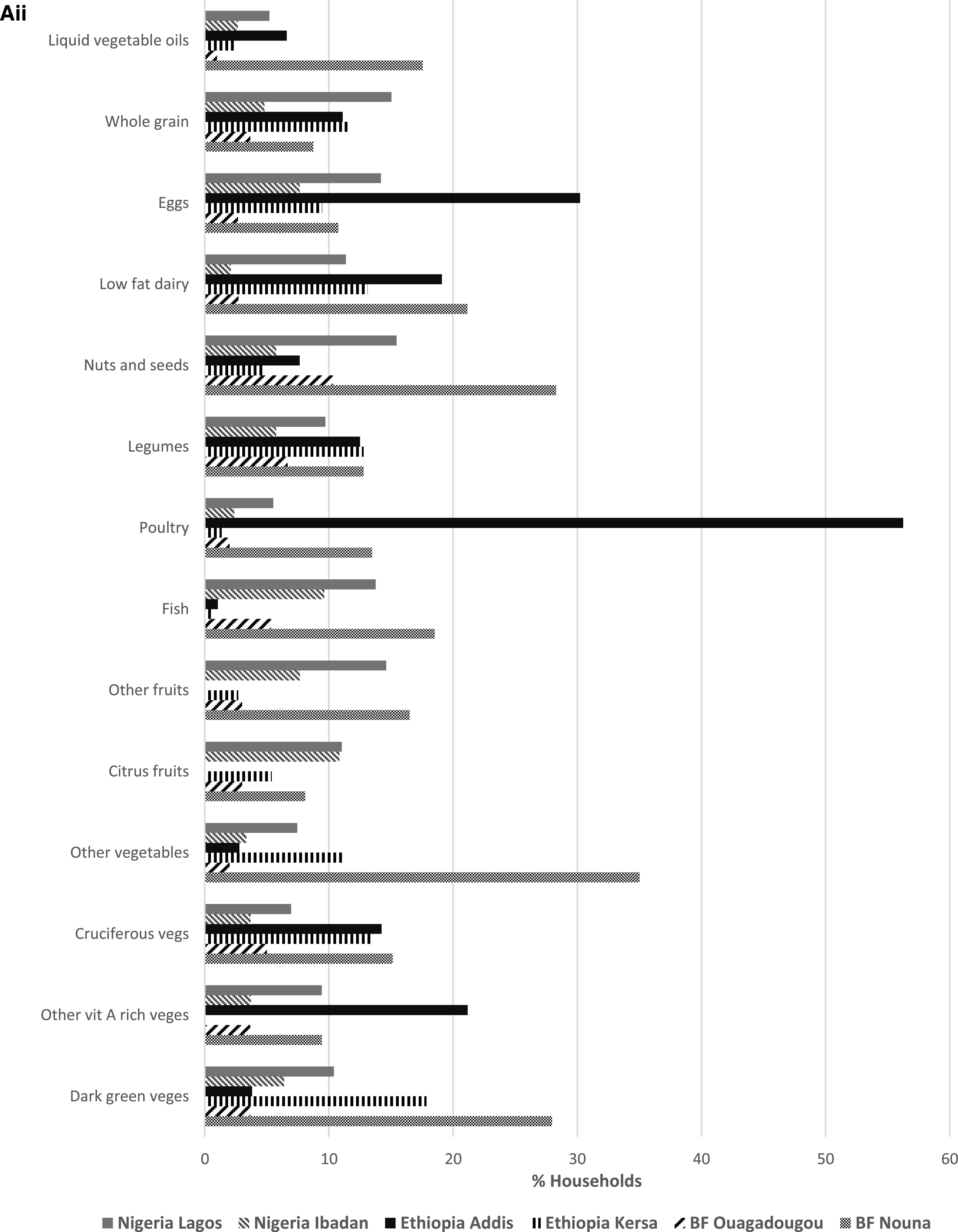
(**A**) Changes in the consumption of healthy Prime Diet Quality Scores (PDQS) food groups before and during the coronavirus disease 2019 (COVID-19) pandemic in Burkina Faso, Ethiopia, and Nigeria. (**Ai**) Decreasing consumption of healthy PDQS food groups in Burkina Faso, Ethiopia, and Nigeria during the COVID-19 pandemic. (**Aii**) Increasing consumption of healthy PDQS food groups in Burkina Faso, Ethiopia, and Nigeria during the COVID-19 pandemic.

In Lagos, at least half of all respondents indicated that consumption of all healthy food groups had declined, with reductions up to 71.4% for poultry and 66.3% for legume consumption compared with that during pre-COVID-19 times. In Ibadan, at least 50% of the households reported that consumption of healthy (other vitamin A-rich vegetables, citrus fruits, fish, poultry, legumes, and eggs) and unhealthy (red meats and refined grains) food groups had also decreased during the same time period. In Kersa, decreases in cruciferous and dark green leafy vegetables consumption were reported by at least one-quarter of the households. In Addis Ababa, at least 40% of households reported decreases in the consumption of cruciferous vegetables and legumes but increased consumption of red meats (45.1%) and poultry (56.3%) during the same time period. Finally, in Nouna, at least 40% of the respondents reported decreased consumption of other vitamin A-rich vegetables, cruciferous vegetables, poultry, and legumes. Red meat consumption also decreased similarly in Nouna (Figure 2Bi). Consumption patterns remained mostly unchanged during the COVID-19 pandemic in Ouagadougou. Figure 3 shows the mean DDS for each study site prior to the COVID-19 pandemic and during the time of the pandemic. The DDS was lower in all sites, except Kersa and Ouagadougou, during the COVID-19 emergency.

**Figure 2B.**
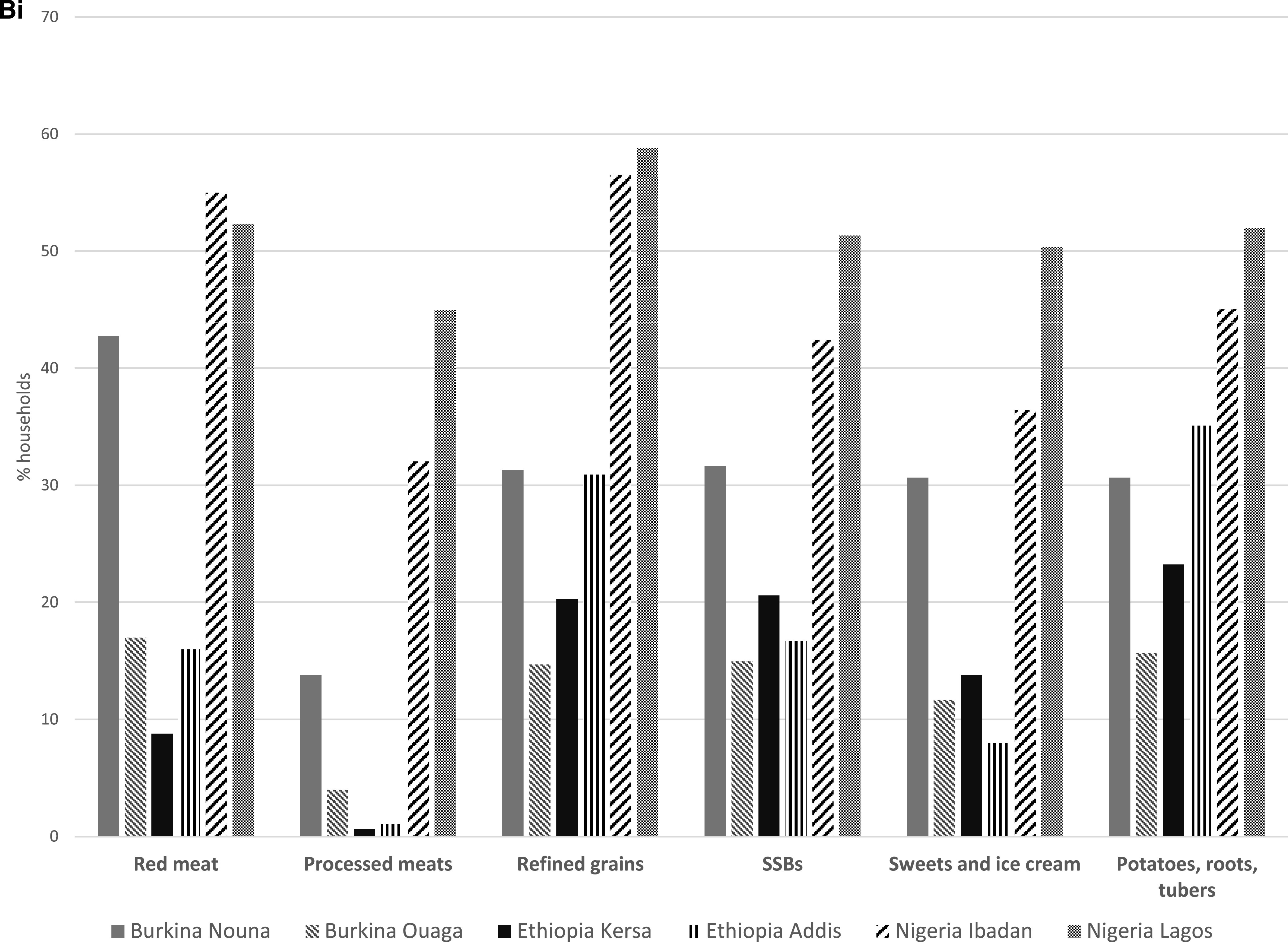

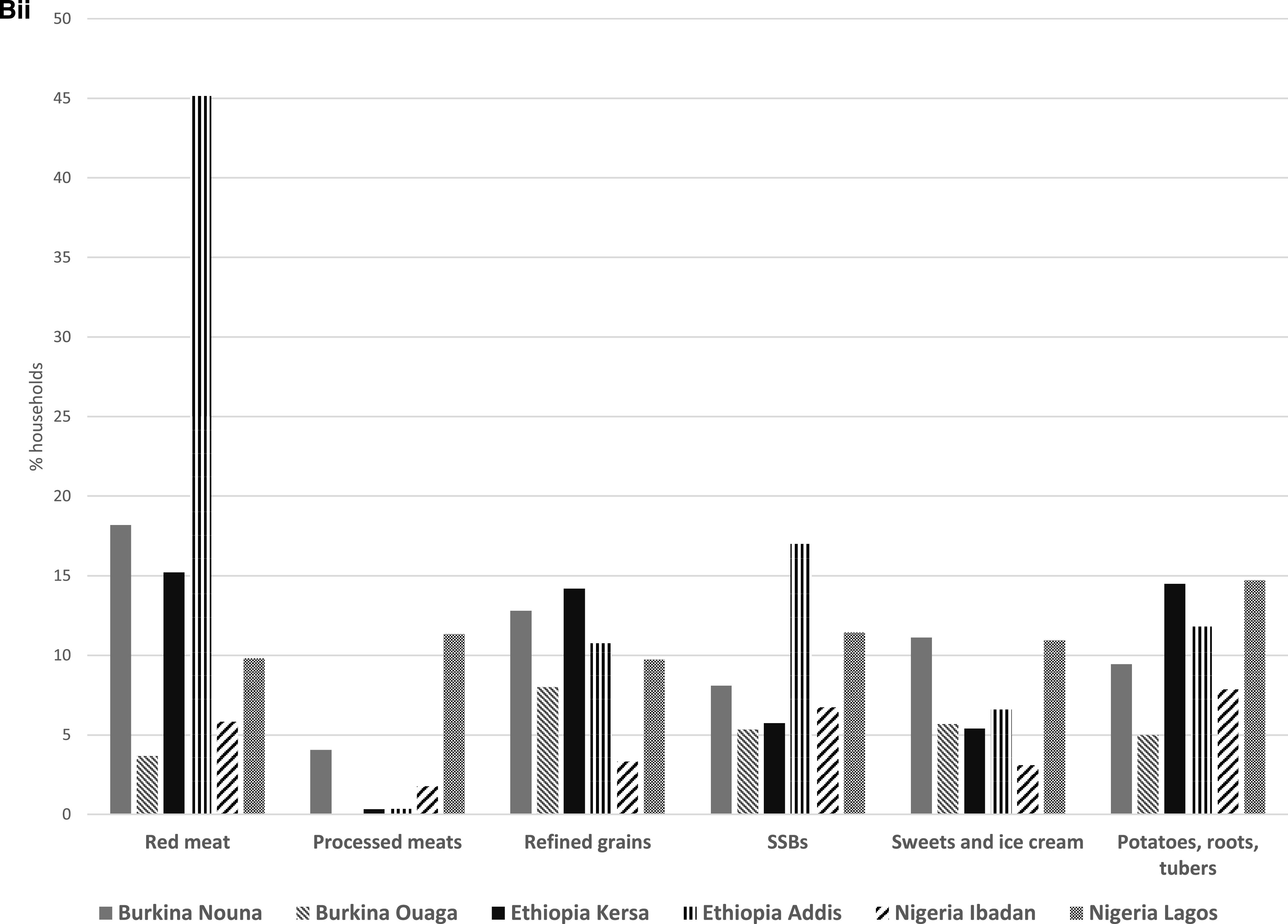
**(B)** Consumption of unhealthy PDQS food groups before and during the COVID-19 pandemic in Burkina Faso, Ethiopia, and Nigeria. **(Bi)** Decreasing consumption of unhealthy PDQS food groups in Burkina Faso, Ethiopia, and Nigeria during the COVID-19 pandemic. **(Bii)** Increasing consumption of unhealthy PDQS food groups in Burkina Faso, Ethiopia, and Nigeria during the COVID-19 pandemic

**Figure 3.**
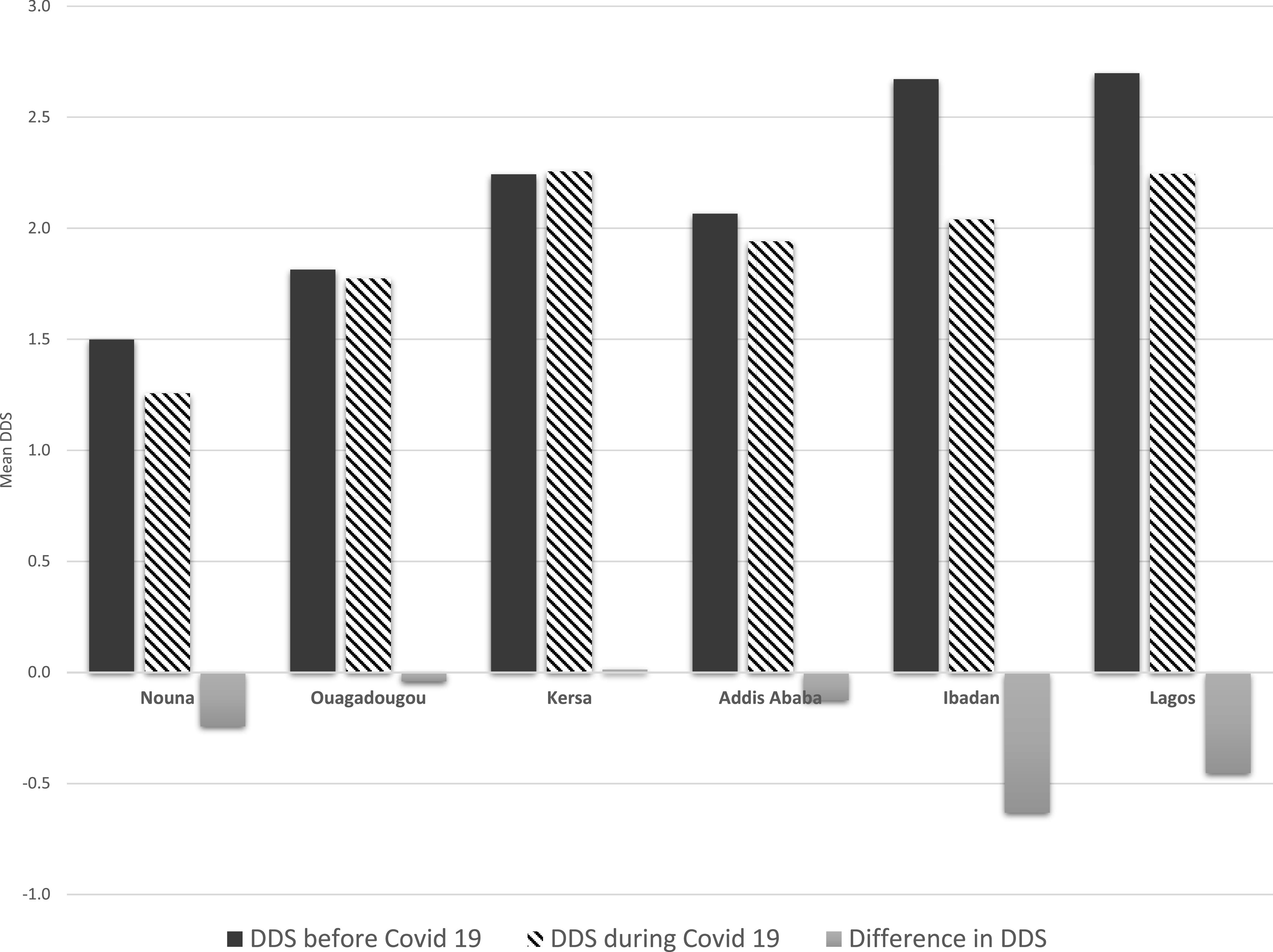
Mean dietary diversity score (DDS) before the coronavirus disease 2019 (COVID-19) pandemic and during the pandemic in Burkina Faso, Ethiopia, and Nigeria. (**A**) DDS based on the consumption of 10 food groups (based on the Minimum Dietary Diversity for Women [MDD-W] food groups). (**B**) For Kersa, the dietary intake excludes other vitamin A-rich fruits and vegetables group. For Addis Ababa, the dietary intake excludes citrus fruits and other fruits groups.

### Changes in food prices and other predictors of dietary diversity and quality.

We evaluated the association of the increase in food prices with DDS during the COVID-19 pandemic in the combined models for all countries (Table 3) and country-specific models (Table 4). We found no overall associations among changes in the prices of staples, fruits, and ASFs and dietary diversity for men and women. However, we found that an increase in the price of pulses was associated with a lower DDS (multivariate-adjusted estimate, −0.35; 95% CI, −0.74 to 0.03; *P* = 0.07) (Table 3) according to the combined analysis. An increase in the price of vegetables was associated with a higher DDS (multivariate-adjusted estimate, 0.22; 95% CI, 0.08 to 0.37; *P* < 0.01). Lower crop production (multivariate-adjusted estimate, −0.54; 95% CI, −0.80 to −0.27) and not engaging in farming (multivariate-adjusted estimate, −0.72, 95% CI: −1.16 to −0.27) were associated with lower DDS according to the combined analysis. Finally, being a farmer or a casual laborer (multivariate-adjusted estimate, −0.44; 95% CI, −0.87 to −0.01) was associated with a lower DDS than being employed (Table 3).

**Table 3:** Association of increase in food prices with the DDS for men and women during the COVID-19 emergency in Burkina Faso, Ethiopia and Nigeria rural and urban sites

	Univariate	Multivariate†
Staple prices		
No change or decreased	ref	ref
Increased	0.03 (−0.13 to 0.19)	0.21 (−0.17 to 0.59)
Pulse prices		
No change or decreased	ref	ref
Increased	−0.14 (−0.44 to 0.16)	−0.35 (−0.74 to 0.03)
Fruits prices		
No change or decreased	ref	ref
Increased	0.03 (−0.14 to 0.19)	−0.04 (−0.16 to 0.07)
Vegetables prices		
No change or decreased	ref	ref
Increased	0.13 (−0.06 to 0.32)	0.22 (0.08 to 0.37)**
Animal source foods prices		
No change or decreased	ref	ref
Increased	0.05 (−0.08 to 0.17)	0.05 (−0.11 to 0.20)
Own crop production affected		
Unchanged	ref	ref
Production has decreased	−0.58 (−0.85 to −0.31)***	−0.54 (−0.80 to −0.27)***
Production has increased	0.17 (−0.15 to 0.49)	0.14 (−0.28 to 0.55)
Does not farm	−0.65 (−1.07 to −0.23)**	−0.72 (−1.16 to −0.27)**
Food security		
Worried you would run out of food (past month)		
No	ref	
Yes	0.05 (−0.39 to 0.48)	
Skipped a meal (past month)		
No	ref	ref
Yes	−0.45 (−0.85 to −0.05)*	−0.33 (−0.74 to 0.08)
Went without eating for a whole day (past month)		
No	ref	ref
Yes	−0.43 (−0.76 to −0.11)*	−0.21 (−0.45 to 0.03)
Age, years		
20–29	ref	ref
30–39	−0.17 (−0.29 to −0.04)*	−0.09 (−0.27 to 0.08)
≥ 40	−0.16 (−0.33 to 0.00)*	−0.09 (−0.30 to 0.12)
Respondent		
Female	ref	ref
Male	−0.01 (−0.14 to 0.11)	0.03 (−0.12 to 0.17)
Education		
None or incomplete primary	−0.11 (−0.35 to 0.12)	0.00 (−0.14 to 0.14)
Primary school or incomplete secondary	ref	ref
Secondary school or higher	0.23 (−0.10 to 0.57)	0.13 (−0.12 to 0.39)
Household head	−0.19 (−0.29 to −0.09)***	−0.15 (−0.34 to 0.12)
Household size	−0.01 (−0.03 to 0.02)	−0.01 (−0.02 to 0.01)
Occupation		
Unemployed	−0.35 (−0.90 to 0.20)	−0.30 (−0.78 to 0.19)
Farmer or casual labor	−0.48 (−1.08 to 0.12)	−0.44 (−0.87 to −0.01)*
Employed	ref	ref
Student, self-employed, or other	−0.27 (−0.68 to 0.13)	−0.24 (−0.54 to 0.07)
Rural	−0.14 (−0.67 to 0.40)	−0.27 (−0.69 to 0.15)
Urban	ref	ref

COVID-19 = coronavirus disease 2019; DDS = dietary diversity score.

* *P* < 0.05, ** *P* < 0.01, *** *P* < 0.001. Combined model for Burkina Faso, Ethiopia, and Nigeria rural and urban sites. Generalized estimating equation (GEE) linear models with exchangeable correlation were used, controlling for clustering by site.

† The multivariate model included covariates significant at *P* < 0.20 in the univariate models. The models evaluated the association of changes in prices for staples, legumes, fruits, vegetables, and animal source foods with the DDS. Models were adjusted for food security (skipped a meal during the past month: no/yes), went a whole day without eating (no/yes), age (20–29, 30–39, ≥ 40 years), respondent sex (female/male), education (none or incomplete primary, primary school or incomplete secondary, secondary school or higher), household head (no/yes), occupation (unemployed, farmer or casual labor, employed, student, self-employed, or other), own crop production affected (unchanged, production has decreased, production has increased, not engaged in farming), and rural location. We forced food production and household size into the multivariate model.

**Table 4:** Association of increases in food prices with the DDS for men and women during the COVID-19 emergency in rural and urban sites in Burkina Faso, Ethiopia, and Nigeria (country-specific models)

	Burkina Faso		Ethiopia		Nigeria	
	Univariate	Multivariate	Univariate	Multivariate	Univariate	Multivariate
Staple prices						
No change	ref	ref	ref	ref	ref	ref
Increased	0.13 (−0.13 to 0.40)	0.34 (−0.04 to 0.72)	0.06 (−0.21 to 0.32)	−0.06 (−0.39 to 0.27)	0.15 (−0.38 to 0.69)	−0.22 (−1.01 to 0.58)
Pulse prices						
No change	ref	ref	ref	ref	ref	ref
Increased	−0.21 (−0.46 to 0.04)	−0.47 (−0.82 to −0.11)*	0.03 (−0.21 to 0.27)	−0.26 (−0.56 to 0.05)	0.07 (−0.45 to 0.58)	−0.33 (−1.15 to 0.50)
Fruits prices						
No change	ref	ref	ref	ref	ref	ref
Increased	0.07 (−0.16 to 0.31)	0.02 (−0.28 to 0.33)	0.16 (−0.03 to 0.34)	−0.12 (−0.36 to 0.11)	0.10 (−0.40 to 0.59)	0.35 (−0.47 to 1.17)
Vegetables prices						
No change	ref	ref	ref	ref	ref	ref
Increased	0.2 (−0.04 to 0.44)	0.08 (−0.26 to 0.42)	0.28 (0.06–0.50)*	0.29 (0.02–0.56)*	0.15 (−0.38 to 0.69)	0.04 (−1.06 to 1.14)
Animal source foods prices						
No change	ref	ref	ref	ref	ref	ref
Increased	0.03 (−0.21 to 0.27)	0.00 (−0.30 to 0.30)	0.25 (0.03–0.47)*	0.21 (−0.07 to 0.49)	0.18 (−0.35 to 0.71)	0.22 (−0.66 to 1.09)
Own crop production affected						
Unchanged	ref	ref	ref	ref	ref	ref
Production has decreased	−1.08 (−1.43 to −0.73)*	−0.72 (−1.07 to −0.38)***	−0.33 (−0.56 to −0.10)*	−0.31 (−0.54 to −0.08)*	−0.51 (−1.32 to 0.31)	−0.41 (−1.19 to 0.38)
Production has increased	−0.37 (−0.72 to −0.01)*	−0.27 (−0.66 to 0.12)	−0.19 (−0.67 to 0.29)	−0.18 (−0.65 to 0.29)	1.40 (0.46–2.34)**	1.04 (0.14–1.93)*
Does not farm	−0.41 (−0.68 to −0.14)**	−1.12 (−1.44 to −0.80)***	−0.39 (−0.59 to −0.19)***	−0.06 (−0.61 to 0.49)	−0.25 (−0.78 to 0.28)	−0.21 (−0.74 to 0.32)
Food security						
Worried you would run out of food (past month)						
No	ref	ref	ref		ref	ref
Yes	0.71 (0.49–0.94)***	0.55 (0.33–0.76)***	−0.04 (−0.21 to 0.13)		−0.45 (−0.75 to −0.15)**	−0.13 (−0.43 to 0.18)
Skipped a meal (past month)						
No	ref		ref	ref	ref	ref
Yes	0.17 (−0.12 to 0.47)		−0.21 (−0.43 to 0.00)	−0.09 (−0.33 to 0.16)	−0.98 (−1.26 to −0.69)***	−0.78 (−1.08 to −0.48)***
Went without eating for a whole day (past month)						
No	ref		ref	ref	ref	ref
Yes	−0.21 (−0.58 to 0.17)		−0.34 (−0.62 to −0.05)*	−0.17 (−0.50 to 0.16)	−0.62 (−0.95 to −0.30)***	−0.33 (−0.64 to −0.01)*
Age, years						
20–29	ref	ref	ref	ref	ref	ref
30–39	−0.04 (−0.72 to 0.63)	0.16 (−0.47 to 0.79)	0.03 (−0.20 to 0.26)	0.07 (−0.17 to 0.32)	−0.31 (−0.77, to 0.15)	−0.30 (−0.73 to 0.14)
≥ 40	−0.06 (−0.69 to 0.57)	0.10 (−0.51 to 0.72)	0.10 (−0.14 to 0.33)	0.16 (−0.09 to 0.42)	−0.31 (−0.71 to 0.08)	−0.32 (−0.72 to 0.07)
Respondent						
Female	ref	ref	ref	ref	ref	ref
Male	−0.02 (−0.3 to 0.26)	0.07 (−0.23 to 0.38)	0.08 (−0.09 to 0.24)	−0.04 (−0.26 to 0.17)	−0.02 (−0.31 to 0.26)	0.09 (−0.25 to 0.43)
Education						
None or incomplete primary	−0.32 (−0.59 to −0.05)*	−0.03 (−0.30 to 0.24)	−0.07 (−0.27 to 0.13)	−0.17 (−0.37 to 0.03)	0.74 (−0.27 to 1.75)	0.67 (−0.29 to 1.63)
Primary school or incomplete secondary	ref	ref	ref	ref	ref	ref
Secondary school or higher	0.18 (−0.58 to 0.95)	0.01 (−0.7 to 0.72)	−0.25 (−0.49 to −0.00)*	−0.22 (−0.48 to 0.03)	0.62 (0.24–0.99)**	0.35 (−0.04 to 0.74)
Household head	−0.09 (−0.43 to 0.25)	−0.26 (−0.62 to 0.11)	−0.13 (−0.34 to 0.08)	−0.11 (−0.36 to 0.13)	−0.21 (−0.49 to 0.07)	−0.23 (−0.58 to 0.11)
Household size	−0.02 (−0.04 to 0.01)	0.00 (−0.02 to 0.03)	0.04 (0.01 to 0.08)*	0 (−0.04 to 0.04)	−0.03 (−0.09 to 0.03)	0.01 (−0.05 to 0.07)
Occupation						
Unemployed	−0.46 (−0.92 to 0.01)	−0.66 (−1.14, to −0.18)*	0.31 (0.04–0.57)*	0.18 (−0.10 to 0.46)	−1.02 (−2.03 to −0.02)*	−0.74 (−1.71 to 0.24)
Farmer or casual labor	−0.77 (−1.13 to −0.41)***	−0.84 (−1.27 to −0.42)***	0.48 (0.25–0.71)***	0.16 (−0.25 to 0.57)	0.51 (−0.18 to 0.51)	0.51 (−0.2 to 1.22)
Employed	ref	ref	ref	ref	ref	ref
Student, self-employed, or other	0.09 (−0.29 to 0.47)	−0.14 (−0.52 to 0.24)	0.30 (0.02–0.57)*	0.25 (−0.02 to 0.52)	−0.68 (−0.97 to −0.39)***	−0.48 (−0.78 to −0.19)**
Rural area	−0.52 (−0.74 to −0.29)***	−0.52 (−0.89 to −0.16)*	0.31 (0.15–0.48)***	0.31 (−0.24 to 0.87)	−0.20 (−0.49 to 0.08)	−0.06 (−0.35 to 0.24)
Urban	ref	ref	ref	ref	ref	ref

COVID-19 = coronavirus disease 2019; DDS = dietary diversity score.

* *P* < 0.05, ** *P* < 0.01, *** *P* < 0.001. Separate models for rural and urban sites in Burkina Faso, Ethiopia, and Nigeria. Generalized estimating equation (GEE) linear models with exchangeable correlation were used.

† The multivariate model included covariates significant at *P* < 0.20 in the univariate models. The models evaluated the association of changes in prices for staples, legumes, fruits, vegetables, and animal source foods with the DDS. Models were adjusted for food security (skipped a meal during the past month: (no/yes), went a whole day without eating (no/yes), age (20–29, 30–39, ≥ 40 years), respondent sex (female/male), education (none or incomplete primary, primary school or incomplete secondary, secondary school or higher), household head (no/yes), occupation (unemployed, farmer or casual labor, employed, student, self-employed, or other), own crop production affected (unchanged, production has decreased, production has increased, not engaged in farming), and rural location We forced food production and household size into the multivariate model.

In country-specific models, we found that in Burkina Faso, an increase in the price of pulses was associated with a lower DDS (multivariate-adjusted estimate, −0.47; 95% CI, −0.82 to −0.11) during the COVID-19 emergency (Table 4). Similar associations with lower agricultural production were observed in Burkina Faso (multivariate-adjusted estimate, −0.72; 95% CI, −1.07 to −0.38) and Ethiopia (multivariate-adjusted estimate, −0.31; 95% CI, −0.54 to −0.08) (Table 4). In Nigeria, skipping a meal (Nigeria) or not eating for an entire day during the previous 30 days were associated with lower dietary diversity (Table 4). Finally, residing in a rural area was associated with consumption of less diverse diets in Burkina Faso (multivariate-adjusted estimate, −0.52; 95% CI, −0.89 to −0.16).

In secondary analysis, we found found no significant associations between increases in the prices of staples, pulses, fruits, vegetables, and ASFs and the PDQS during the COVID-19 pandemic in adjusted models (Supplemental Table 2). Lower crop production (multivariate-adjusted estimate, −1.13; 95% CI, −1.85 to −0.40) and not engaging in farming (multivariate-adjusted estimate, −1.68; 95% CI, −2.04 to −1.33) were associated with lower PDQS during the COVID-19 pandemic. Not eating for a whole day during the past month (multivariate-adjusted estimate, −0.85; 95% CI, −1.32 to −0.38) was associated with a lower PDQS. Being unemployed (multivariate-adjusted estimate, −0.97; 95% CI, −1.76 to −0.18), being a farmer or a casual laborer (multivariate-adjusted estimate, −1.13; 95% CI, −2.24 to −0.02), and being self-employed or a student (multivariate-adjusted estimate, −0.83; 95% CI, −1.97 to −0.20) were associated with lower PDQS than being employed.

## DISCUSSION

Overall, we found that in all SSA sites evaluated during this study, most respondents reported that prices for staples, pulses, fruits, vegetables and ASFs had increased during the COVID-19 emergency. Additionally, dietary diversity and quality decreased modestly compared to pre-COVID-19 times across most sites. We found that increases in the price of pulses were associated with lower DDS. We also found a significant association between increases in the prices of vegetables and higher DDS overall. However, we found no significant association between increases in the prices of key food groups and diet quality for participants.

The impact of the COVID-19 crisis on food security and nutrition in low-income and middle-income countries is an area of continued research. In our study, we found that all sites reported that prices of key food groups had increased during the COVID-19 pandemic, with most notable increases observed in Ethiopia and Nigeria. These findings are consistent with the projections of previous studies. Early estimates anticipated that the effects of COVID-19 on food systems in low-income and middle-income countries would include disruptions in food supplies as a result of restrictions on the movement of people, export restrictions that disrupted trade flows and supply chains including for staple foods (such as wheat and rice), economic downturn and loss of income.^15,27^ The impacts of these were expected to include decreased availability of food and increased food prices, resulting in lower access to food and shifts in consumer demand toward cheaper and less nutritious foods.^15^ We believe that disruptions in the availability and affordability of nutritious food during our study could have occurred because of various reasons. The causes of disruptions in the supply chain are likely to vary in different contexts (e.g., rural compared with urban locations). In some locations, particularly in urban areas, restrictions caused by the lockdowns and related disrupted physical mobility (and market closures) and job losses may have more importantly affected dietary diversity. Alternatively, in rural areas, disruptions to food transport or the lack of means to transport food commodities for sale would have led to losses for farmers. Additionally, limited access to inputs (e.g., seeds and fertilizers) would have decreased production.

A recent study conducted in Zimbabwe found that COVID-19-related lockdowns led to increased food prices for 95% and decreased availability of nutritious foods for 64% of the respondents.^28^ This is consistent with the results of a previous study performed during the global food crisis in 2008 that reported that the food crisis led to increased prices for fish (113%), cereals (53%), and vegetable oil (44%) in local markets in Burkina Faso.^29^ Therefore, our findings of potential increases in food prices are plausible. It is important to note that although increases occurred across all sites, the extent of the increases was more severe in Nigeria and Kersa.

Overall, this study showed decreased consumption of diversified diets during the COVID-19 pandemic, with the consumption of staples, legumes, and other vegetables decreasing for at least 40% of all households across all sites except Kersa and Ouagadougou. In Burkina Faso, we found that although consumption of staples, legumes, fruits, vegetables, and meats decreased, along with dietary diversity in Nouna, consumption was relatively unchanged in Ouagadougou during the COVID-19 crisis. For the PDQS food groups, decreases were noted, although to a lesser extent across sites. It was notable that in Lagos, more than 50% of the respondents reported decreased consumption of all healthy food groups for the PDQS. Conversely, the consumption of poultry, eggs, and red meat was reportedly higher in Addis Ababa compared with the period before COVID-19.

Our findings of decreasing overall dietary diversity and quality at most sites were consistent with our expectations. However, decreases in DDS and PDQS were small in Ethiopia. This is consistent with a recent study performed in Addis Ababa that reported that despite previous studies documenting extensive losses of incomes, food consumption and household dietary diversity were largely unchanged during the COVID-19 pandemic.^30^ This was attributed to the fact that Ethiopia did not have a total lockdown that severely restricted movement like its neighboring countries.^30^

During this study, we found that higher pulse prices were associated with the consumption of less diversified diets in Burkina Faso. Increases in the prices of pulses (which tend to be a cheaper alternative to ASFs, but more expensive than vegetables) are likely to lead to their substitution from the diet, resulting in less diversity in intake. Previous studies suggested that decreasing dietary diversity is a common strategy for households to cope with higher staple food prices, along with changing the quality and quantity of foods consumed.^31,32^ This finding is concerning considering that legumes are an important part of the diet as a source of protein and essential amino acids such as lysine, carbohydrates, dietary fiber, B vitamins such as folate, and minerals such as calcium and zinc.^33^ Therefore, a reduction in the consumption of legumes will negatively impact nutrition for vulnerable households.

We also found that increased prices of vegetables were associated with consumption of more diversified diets during the cross-country analysis and in Ethiopia. A plausible explanation for our findings is that increasing vegetable prices may be beneficial for households selling vegetables because increased income could be used to purchase diets that are more diverse.

We found that crop production was prevalent and farming was a key livelihood in the rural sites of Nouna and Kersa. However, decreases in agricultural production during the COVID-19 pandemic were noted in both areas. A previous study performed in Ethiopia found that COVID-19 disruptions affected vegetable farmers because of limited access to services and the unavailability of on-farm labor, as well as increased production costs and decreased availability of inputs.^34^ The study found increased vegetable prices as a result of lower agricultural production and the need to import foods.^34^ The reported decreases in agriculture production at our study sites could potentially impact food prices in these areas.

We found that decreased crop production was associated with less diverse diets. This finding suggested that crop production may be an important contributor to diversified dietary intake in the countries studied. This is consistent with previous findings that crop production can influence dietary diversity through production diversity and income pathways.^35,36^ The effects of COVID-19 on agriculture production and dietary diversity could be partially attributable to disruptions of supply chains, including for inputs, delayed or lower harvests, damage of perishable produce, and loss of income for farmers.^37^ Telephone surveys were used to evaluate the effects of COVID-19 on food systems in India,^38^ where farmers reported initial disruptions in production, sales, and prices, as well as lower incomes from agriculture because of COVID-19 restrictions. Households reported disruptions to their diets, with decreases in the consumption of fruit and ASFs, excluding dairy, whereas vegetable consumption increased in some households.^38^ A subsequent study indicated that market reforms helped mitigate price increases for vegetables and wheat because of COVID-19 restrictions in this context.^39^ This suggests that policy interventions during the COVID-19 pandemic can influence food prices.

When we evaluated food security during the study, we found that the majority of the households had worried about food during the previous month, many skipped a meal (up to 70% in Ibadan and 59% in Lagos), and 15% did not eat for a whole day during the previous 30 days. We found that food insecurity (skipping a meal and not eating for an entire day) was negatively associated with dietary diversity and quality in Nigeria. A previous study in the same context found that households that had been exposed to lockdowns experienced increased food insecurity, as well as reduced wages and farming activities.^40^ We also found that farming/casual labor was associated with lower dietary diversity and quality compared with formal employment. This may indicate that those who are formally employed may be affected to a lesser extent by lockdowns. Studies show that COVID-19 restrictions tend to have a greater impact on informal employment and casual labor, as job losses are more likely for these sectors, and that those who are casually employed or unemployed may be more vulnerable and have fewer coping strategies.^41^ A study conducted in rural Malawi and Liberia found that market activity was severely disrupted in response to COVID-19 restrictions, and that income decreased, particularly for market vendors.^42^ In Nigeria, COVID-19-related food price increases were associated with food insecurity, and poorer households and those engaged in nonfarm business activities were most affected.^40^ Those findings are consistent with our findings.

There were several strengths to our study. We used a novel design and platform to conduct telephone surveys in multiple countries in SSA (Burkina Faso, Ethiopia, and Nigeria) to generate comparable data regarding the effect of COVID-19 on prices of main food groups (staples, pulses, fruits, vegetables and ASF) and to assess the impact of changes in prices on dietary diversity and quality the context of the COVID-19 emergency. Study limitations included our cross-section study design, our inability to assign causality, and possible residual confounding. We adjusted for socio-economic and other household factors, including education level and the age and sex of the respondent to address issues of potential confounding. Finally, we did not assess MDD-W based on 24-hour dietary recall; therefore, we had to standardize the frequency of consumption over the course of 7 days to consumption in a day. Therefore, the DDS measure may have underestimated the diversity of intake during the study. However, we believe that the decreases in consumption for the DDS were consistent with the observed decreases in the PDQS during the COVID-19 pandemic, as well as the associations observed for food security and other factors.

In conclusion, we observed potential negative effects of COVID-19 (both direct and indirect) on agriculture production, food prices for staples and other nutrient-dense food groups, food security, and dietary diversity and quality in Burkina Faso, Ethiopia, and Nigeria. These factors may impact the nutrition and health of vulnerable groups in these countries and require scrutiny by policymakers and programs in these countries. The price increases and worsening dietary diversity and quality call for social protection and other strategies to increase the availability and affordability of nutrient-rich foods during the COVID-19 pandemic and other public health emergencies. Monitoring and tracking of changes in these factors within and across countries are imperative for informed decision-making and quick responses to address and mitigate potential negative effects on health and nutrition.
